# A Narrative Review of the Association of Obstructive Sleep Apnea with Hypertension: How to Treat Both When They Coexist?

**DOI:** 10.3390/jcm12124144

**Published:** 2023-06-20

**Authors:** Servet Altay, Selma Fırat, Yüksel Peker, The TURCOSACT Collaborators

**Affiliations:** 1 Department of Cardiology, Trakya University School of Medicine, Edirne 22030, Turkey; svtaltay@gmail.com; 2 Department of Pulmonary Medicine, University of Health Sciences, Atatürk Sanatorium Education and Research Hospital, Ankara 06280, Turkey; selmafirat1@gmail.com; 3 Department of Pulmonary Medicine, Koç University School of Medicine, Istanbul 34450, Turkey; 4 Division of Sleep and Circadian Disorders, Brigham and Women’s Hospital, Boston, MA 02115, USA; 5 Division of Pulmonary, Allergy, and Critical Care Medicine, University of Pittsburgh School of Medicine, Pittsburgh, PA 15213, USA; 6 Department of Clinical Sciences, Respiratory Medicine and Allergology, Faculty of Medicine, Lund University, 22002 Lund, Sweden; 7 Department of Molecular and Clinical Medicine, Institute of Medicine, Sahlgrenska Academy, University of Gothenburg, 40530 Gothenburg, Sweden

**Keywords:** obstructive sleep apnea, hypertension, narrative review

## Abstract

Hypertension (HT) is a worldwide public health issue and an essential risk factor for cardiovascular and cerebrovascular diseases. Obstructive sleep apnea (OSA) is a condition characterized by recurrent episodes of apnea and hypopnea as a consequence of partial or complete obstruction of the upper airways due to anatomic and/or functional disturbances. There is mounting evidence of a relationship between OSA and HT. In patients with OSA, HT is predominantly nocturnal and characterized by high diastolic blood pressure and usually by a nondipping pattern. Optimizing the blood pressure control is recommended in the current guidelines as the first treatment option in hypertensive patients with OSA. Continuous positive airway pressure (CPAP) therapy may reduce blood pressure, albeit only slightly as a stand-alone treatment. CPAP, as an add-on treatment to antihypertensive medication, appears to be an efficient treatment modality when both conditions coexist. This narrative review aims to summarize the current perspectives on the association of OSA with HT and the treatment options available for adults with OSA-related HT.

## 1. Introduction

In general, patients with an office systolic blood pressure (SBP) ≥ 140 mm Hg and a diastolic blood pressure (DBP) ≥ 90 mmHg are deemed to have hypertension (HT). The overall prevalence of HT in adults is reportedly around 30–45% [1]. Obstructive sleep apnea (OSA) is a condition with recurrent apnea and hypopnea, frequent arousal, and hypoxemia, which can lead to serious cardiovascular consequences such as HT, heart failure, arrhythmia, and atherosclerosis [2]. As an important cause of morbidity and mortality, HT accounted for the deaths of approximately 10 million people in 2015 and over 200 million disability-adjusted life years [3]. Blood pressure normally drops during sleep. A nocturnal decrease of more than 10% in the mean blood pressure level throughout the day is defined as the “dipping pattern”. The absence of this decrease indicates a nondipping pattern. One of the important causes for the nondipping pattern is OSA [4,5]. Here, the sympathetic nervous system is activated due to the obstructed airway in patients with OSA, resulting in the disruption of the natural dipping pattern and causing an increase in blood pressure [5]. Traditionally recognized risk factors for cardiovascular diseases (CVDs), such as obesity, insulin resistance, diabetes mellitus, and hyperlipidemia, are common in OSA patients, and the most common CVD in patients with OSA is HT. The coexistence of OSA and chronic obstructive pulmonary disease (COPD) is called overlap syndrome [6], and individuals with overlap syndrome have a significantly increased risk of HT compared with those with COPD alone [7].

On behalf of the Turkish Collaboration of Sleep Apnea Cardiovascular Trialists (TURCOSACT), we recently published a position paper on OSA and cardiovascular diseases [8]. To provide in-depth analyses of the topic, the present narrative review aimed to summarize the existing perspectives on the association of OSA with HT and the treatment options available for individuals with OSA and concomitant HT.

## 2. Epidemiology of OSA and Hypertension

HT and OSA often coexist. Pensukan et al. found a significant relationship between OSA and elevated blood pressure (odds ratio (OR): 2.38; 95% confidence interval (CI): 1.68–3.39), and HT (OR: 2.55; 95% CI: 1.57–4.15) after adjusting for demographic characteristics [9]. OSA has been reported among 30–50% of hypertensive patients. This rate can increase to 80% among cases with drug-resistant HT [10,11,12]. It has also been reported that masked HT is 2.7 times more common in OSA patients [13,14].

There is a bidirectional and causal relationship between HT and OSA. Several studies have revealed a clear dose–response relationship with OSA severity and HT. The meta-analyses on the relationship between OSA and HT are summarized in Table 1 [15,16,17,18].

The Sleep Heart Health Study (SHHS), a large-scale, community-based, multicenter, cross-sectional study conducted with 6152 participants, reported an increased OR of 1.37 (95% CI: 1.03–1.83) of HT in those with severe OSA after adjusting for confounding factors [12]. Different results have been reported on the relationship between OSA and HT in prospective studies. One of these studies was the Wisconsin Sleep Cohort Study, which was conducted with 709 participants. The results indicated a dose–response relationship independent of known confounding factors between sleep-disordered breathing and new-onset HT 4 years later [19]. Similarly, in the Zaragoza Sleep Cohort Study, a prospective, observational study conducted with 1889 participants for a mean follow-up duration of 12.2 years, an increased risk of new-onset HT was detected in untreated OSA patients after adjusting for confounding factors, including apnea hypopnea index (AHI), age, sex, baseline SBP, DBP, and body mass index (BMI) [20].

In contrast, in the Victoria sleep cohort study conducted with 1557 participants for a follow-up duration of 7.5 years, no relationship was found between OSA and the incidence of HT [21]. Along these lines, the 5-year follow-up study of the SHHS, conducted with 2470 participants without HT at admission, found that after adjusting for BMI, AHI was no longer a significant predictor of HT. The findings that do not support the relationship between OSA and HT were attributed to the lower rate of participants with moderate-to-severe OSA. Indeed, the vast majority (around 87%) of the participants included in the 5-year follow-up of the SHHS had mild OSA, defined as an AHI between 5 and 15 events/h [22].

A meta-analysis of seven studies by Xia W et al., published in 2018, including 6 prospective cohort studies and 1 case-control study, conducted with a total of 6098 participants, reported that high AHI values were related to a significantly increased risk of essential HT compared with low AHI values (OR 1.77, 95% CI 1.30–2.41, *p* = 0.001). The results of the linear dose–response meta-analysis indicated that the risk of essential HT increased by 17% for every 10 events/hour increase in the AHI (OR 1.17, 95% CI 1.07–1.27, *p* = 0.001). Moreover, the nonlinear dose–response found in the meta-analysis results revealed that the risk of essential HT increased with AHI [23]. Similarly, a recent meta-analysis by Yuan F. et al. published in 2021, including 8 studies conducted with a total of 3484 OSA patients, revealed a significant association between OSA and HT (OR 6.44, 95% CI 5.38–7.71, *p* < 0.001) and between OSA severity and HT [17].

## 3. Pathogenesis of Hypertension in OSA

**Figure 1:**
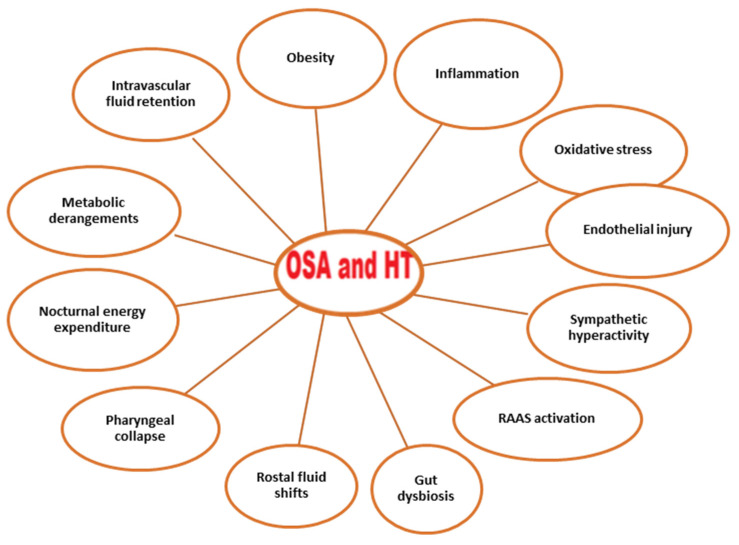
Conditions associated with OSA and hypertension. Abbreviations: OSA, obstructive sleep apnea; RAAS, renin–angiotensin–aldosterone system.

The mechanisms promoting HT in OSA are multifactorial. Sympathetic activity due to intermittent hypoxia is one of the mechanisms triggering the elevation in blood pressure in OSA. Sympathetic activity due to the hypoxemic state causes both vasoconstriction and the stimulation of chemoreceptors. Consequently, the renin–angiotensin–aldosterone system (RAAS) is activated, the endothelin-1 level is increased, and the nitric oxide level is decreased, all of which contribute to the increase in vascular resistance and the development of HT [24,25]. In addition, RAAS activation increases the amount of angiotensin-2, a strong vasoconstrictor, in the blood and, thus, blood pressure [26]. Increased aldosterone levels also contribute to the development of HT by causing fluid and sodium retention [27]. Sympathetic hyperactivity leads to a proinflammatory state, resulting in endothelial injury and oxidative stress [28,29]. The other factors that play a role in the pathogenesis of HT in OSA are obesity, gut dysbiosis, rostral fluid shifts, pharyngeal collapse, nocturnal energy expenditure, and metabolic derangements [24] (Figure 1).

## 4. Clinical Characteristics of Hypertension in OSA

In patients with OSA, HT is predominantly nocturnal and characterized by a high DBP, masked HT, and a nondipping pattern [4]. Blood pressure is normally the highest during the mid-morning, gradually decreasing as the day progresses, down to 10% of the wakefulness value during sleep, and reaches its lowest value at 3 a.m. This dipping pattern is correlated with the duration of deep sleep. It has been reported that the expected decrease in blood pressure may not occur in the case of several diseases such as OSA. Important cardiovascular consequences may occur in patients who have such diseases who are referred to as “nondippers” [30,31].

Nocturnal blood pressure elevation is correlated with the severity of OSA [32]. The nondipping blood pressure pattern was reported at a rate of 84% in OSA patients who did not receive treatment [33]. Nocturnal and nondipping HT is closely associated with target organ damage and the development of cardiovascular diseases [34]. Additionally, it has been reported that night-time blood pressure variability, which is related to increased target organ damage, was higher in a OSA group than in a non-OSA group [35,36]. The pathogenesis of the nondipping pattern and blood pressure variability is multifactorial. Intermittent hypoxia and recurring microarousals are major events leading to sleep fragmentation, reduced slow-wave sleep, and increased sympathetic activity, resulting in elevated blood pressure and increased blood pressure variability [37].

## 5. Treatment Modalities

Among the treatment modalities that come to the fore in the treatment of OSA in patients with HT are CPAP, diuretics, renal denervation, use of maxillomandibular advancement devices, and hypoglossal nerve stimulation surgery for restricted airways or tonsillar enlargement. Weight loss, physical exercise, reducing alcohol consumption, and smoking cessation are among the primary lifestyle changes recommended for hypertensive patients with OSA [38].

## 6. Pharmacological Therapies of HT in Patients with OSA

### 6.1. Antihypertensive Medications

Current guidelines for HT do not make specific recommendations on the pharmacological treatment modalities for patients with concomitant OSA and HT. Given the increased sympathetic activity and renin–angiotensin–aldosterone (RAAS) activity in OSA patients, medications that block these pathways are highlights. Among the antihypertensive medications that were initially preferred were angiotensin-converting enzyme (ACE) inhibitors and angiotensin receptor blockers (ARBs) [39]. It was also demonstrated that beta-blockers mitigate night-time blood pressure rise and apnea-related tachycardias [40]. An earlier study conducted by Hedner et al. compared the effects of atenolol, hydrochlorothiazide, amlodipine, enalapril, and losartan on office and ambulatory blood pressures in 40 individuals with HT and OSA [41]. Each participant received two of the aforementioned five agents (balanced incomplete block design) for 6 weeks, with a 3-week washout period in-between. Compared with the other four drugs, atenolol lowered the office diastolic BP as well as mean night-time ambulatory SBP and DBP [41]. These findings support the hypothesis that overactivity of the sympathetic nervous system is the most important mechanism involved in the development of HT in adults with OSA [41].

### 6.2. Diuretics

Two small studies have suggested that the use of spironolactone, an aldosterone antagonist, may efficiently decrease blood pressure in OSA patients with treatment-resistant HT [42,43]. Similarly, eplerenone, another aldosterone antagonist, was shown to significantly decrease blood pressure in hypertensive OSA patients [44]. Recent studies have shown that primary aldosteronism is common in patients with moderate-to-severe OSA, a finding that indicates aldosterone antagonists may be beneficial in this patient group [45,46,47]. There are also studies suggesting that aldosterone antagonists may reduce the frequency of apnea by mitigating laryngeal edema in OSA patients [42,46]. Taken together, these findings suggest that aldosterone antagonist diuretics, especially spironolactone, may be effective in the treatment of HT in OSA patients. However, large-scale cohort studies are needed to elucidate the efficacy of aldosterone antagonist diuretics in treating HT in OSA patients.

### 6.3. Sodium-Glucose Cotransporter 2 (SGLT2) Inhibitors

Canagliflozin, one of the SGLT2 inhibitors that has recently been the focus in the treatment of cardiac failure, was shown to provide significant nocturnal blood pressure reductions in adults with diabetes, treatment-resistant HT, and OSA [48].

## 7. CPAP Therapy for OSA in Patients with HT

A number of studies have shown that CPAP therapy results in a modest reduction of 2–3 mmHg in SBP and of 1.5–2 mmHg in DBP in OSA patients (Table 2) [49,50,51,52,53,54,55,56]. On the other hand, this reduction is higher in adults with treatment-resistant HT. Although a 1–2 mmHg decrease in blood pressure may not be considered much, even such a slight decrease in blood pressure was shown to be associated with significant decrease sin cardiovascular mortality and stroke risk [57].

The efficacy of CPAP therapy in the treatment of HT in patients with OSA has been extensively investigated in the literature. Earlier meta-analyses demonstrated a good effect of CPAP therapy in lowering blood pressure in OSA patients. Studies have shown significant reductions in day- and night-time blood pressure values, especially in OSA patients with treatment-resistant HT [58,59,60,61].

Kartali N. et al. reported that SBP decreased from 141.5 ± 12.1 mmHg to 133.5 ± 9.7 mmHg (*p* = 0.007) and that DBP decreased from 87.8 ± 6.8 to 83 ± 5.4 mmHg (*p* = 0.004) after three months of CPAP treatment [62]. Interestingly, the decrease in SBP was observed only during the night (*p* = 0.031), whereas the decrease in DBP was both during the day and night (*p* = 0.024 and *p* = 0.007, respectively). Of note, all the hypertensive participants included in the study were initially nondippers, and the dipping status was significantly improved after CPAP therapy (from 7.9 to 10.4% for SBP, *p* = 0.014; and from 8.4 to 10.5% for DBP, *p* = 0.029) [62].

In a randomized controlled trial (RCT) by Hoyos C. et al., a significant decrease was detected in blood pressure after CPAP therapy. The magnitude of the decrease was 4.1 mmHg regarding the mean central SBP (*p* = 0.003), 3.9 mmHg in mean central DBP (*p* = 0.0009), 4.1 mmHg in mean peripheral SBP (*p* = 0.004), and 3.8 mmHg in mean peripheral DBP (*p* = 0.001) [63].

In a four-year retrospective study by Yang M.C. et al., the mean blood pressure decreased from 100.8 ± 13.6 mmHg to 96.6 ± 10.8 mmHg (*p* = 0.004) in OSA patients who were adherent with CPAP [64].

A 24-week follow-up study by Campos-Rodriguez F. et al. demonstrated that dose-related beneficial effects were achieved in the long term, even in hypertensive OSA patients who were initially undertreated [65].

In an RCT comparing three months of CPAP therapy with sham-CPAP in adults with moderate-to-severe OSA and nocturnal HT, a slight decrease in 24 h SBP/DBP by 2.8/2.5 mmHg was observed, but this was not statistically significant [66]. The blood-pressure-lowering effect of CPAP was shown to depend on the baseline daytime pulse rate; and, accordingly, the reduction was significantly greater (10.1 mmHg or more) in patients with a greater daytime pulse rate [66].

A multicenter RCT comparing CPAP with sham-CPAP in 272 patients with new-onset systemic HT and moderate-to-severe OSA revealed a significant effect of CPAP on the dipping pattern [65]. CPAP therapy was associated with reductions in 24 h ambulatory blood pressure variables and night-time ambulatory blood pressure measurements only in the nondipper group, whereas no significant difference was detected in the dipper group [67].

In a recent 8-week-long parallel-group RCT conducted with 92 patients with treatment-resistant HT and OSA, the participants were randomized to CPAP and no-CPAP groups [68]. Significant decreases were observed in 24 h SBP by 4.4 mmHg, in 24 h DBP by 2.9 mmHg, in daytime SBP by 5.4 mmHg, and in daytime DBP by 3.4 mmHg, yet only in nondippers, not in dippers [68].

Other studies have evaluated the effect of antihypertensive medications vs. CPAP on blood pressure in patients with OSA, suggesting that antihypertensive drugs reduce the blood pressure better than CPAP therapy alone and that the combined use of CPAP therapy and antihypertensive medications provide better results than the stand-alone use of either treatment method [69,70].

The application of CPAP therapy for one more hour per night reportedly reduced SBP and DBP 1.5 mmHg and 0.9 mmHg more, respectively [71]. CPAP therapy reduced blood pressure more in adults with severe OSA and in those with more complaints of insomnia during the day [72].

It has been proposed that the blood-pressure-lowering effect of CPAP therapy is more prominent in patients under 60 years of age, in those with higher pretreatment blood pressure, in untreated HT, treatment-resistant HT, nocturnal or nondipping HT, severe OSA, and in those who are adherent to CPAP therapy [13,50,52,71,73]. Nonetheless, given that CPAP therapy does not correct all factors that increase blood pressure (e.g., volume overload, high salt production, etc.), CPAP alone cannot produce a significant improvement in blood pressure. According to the 2017 American Heart Association (AHA)/American College of Cardiology (ACC) blood pressure guidelines, CPAP therapy is yet not a well-established antihypertensive treatment in adults with HT and OSA (class IIb) [74].

## 8. Non-CPAP Treatments of OSA in Patients with HT

A number of studies, though small-scale and observational, have demonstrated that non-CPAP treatments, e.g., soft-palate lifters, tongue-retaining devices, mandibular advancement appliances, expansion sphincter pharyngoplasty, etc., provided blood pressure reduction comparable to CPAP therapy in patients with HT in patients with OSA [52].

In one of these RCTs, including 65 hypertensive patients with OSA, uvulopalatopharyngoplasty (UPPP) provided a blood pressure reduction of 4–9 mmHg compared with the control group [75]. Previous studies reported that CPAP therapy was not superior to UPPP in preventing the development of hypertension in OSA patients [76]. In contrast, a recent study conducted with 413 OSA patients demonstrated that both CPAP therapy and UPPP had a preventative effect on the development of HT in OSA patients and that CPAP treatment prevented the development of HT more than UPPP [77]. Another recent study revealed that expansion sphincter pharyngoplasty resulted in significant blood pressure reduction in OSA patients [78].

## 9. Renal Denervation for Treatment-Resistant HT in OSA

It has been suggested that renal sympathetic denervation, which has recently emerged as a new approach in the treatment of treatment-resistant HT, might also be used in the treatment of HT in patients with OSA. Daniels et al. conducted a prospective study in patients with OSA and treatment-resistant HT and demonstrated that renal denervation (RDN) provided a significant decrease in office and ambulatory blood pressure values after a six month follow-up period [79]. Similarly, an RCT conducted with moderate-to-severe OSA patients with treatment-resistant HT demonstrated that RDN safely provided significant blood pressure reduction compared with the control group [80]. Further large-scale studies on the efficacy of RDN in OSA patients are needed.

## 10. Summary, Generalization, and Inferences

Based on the current evidence about the association between OSA and HT, it appears appropriate that patients with drug-resistant or poorly controlled or nondipping hypertension should be screened for OSA [8]. Weight loss, regular physical exercise, a healthy diet, and salt restriction are important for blood pressure regulation in patients with OSA and HT. Regarding the medications, calcium channel blockers, ACEIs or ARBs, and thiazide-type diuretics are recommended as first-line therapy in patients with OSA and HT, and spironolactone should be added to the first line treatment in the presence of resistant hypertension in OSA patients. Moreover, beta-blocker therapy should be preferred in hypertensive OSA patients in the presence of CAD, arrhythmia, or heart failure, whereas SGLT-2 inhibitors and renal denervation therapy may be considered as an option in patients refractory to standard therapy. Last but not least, CPAP therapy should be considered at all stages in OSA patients with HT, and it should definitely be used, especially in cases with drug-resistant HT [8].

## 11. Conclusions

OSA and HT often coexist, which means OSA is one of the important factors to be considered in managing treatment-resistant HT. The current guidelines for the treatment of HT in patients with OSA adopt the general principles of HT treatment. Hence, even though it provides a modest blood pressure reduction, CPAP therapy is considered in the treatment modalities recommended for the treatment of HT, especially as an add-on treatment. In this context, high CPAP compliance is crucial for the effective treatment of HT in OSA. The number of studies on the efficacy of pharmacological treatments and surgical treatments is limited. Further large-scale studies featuring conventional and novel treatment approaches are needed to serve as a guide for future studies related to OSA and HT.

## Figures and Tables

**Table 1:** Recent meta-analyses regarding the association of OSA with the risk of HT.

Author (Reference)	Year	Number of Studies	Total Sample Size	OSA (OR (95% CI)) for HT
Meng [15]	2016	6	20,367	1.41 (1.29–1.89)
Hou [16]	2018	26	51,623	1.80 (1.54–2.06)
Han [17]	2020	10	13,274	1.80 (1.36–2.38)
Yuan [18]	2021	8	3484	6.44 (5.38–7.71)

Abbreviations: CI, confidence interval; HT, hypertension; OR, odds ratio; OSA, obstructive sleep apnea.

**Table 2:** Recent meta-analyses regarding the effect of OSA treatment on blood pressure values.

Author (Reference)	Year	Number of Studies	Total Sample Size	OSA Therapy	Main Findings
Liu [49]	2016	5	446	CPAP	MBP reduction: 4.78 mmHg, 95% C:I 1.61–7.95 mmHg SBP reduction: 2.95 mmHg, 95% CI: 0.53–5.37 mmHg DBP reduction: 1.53 mmHg, 95% CI: 0.00–3.07 mmHg
Pengo [50]	2020	68		CPAP or MADs	MBP reduction: 2.09 mmHg, 95% CI: 2.78–1.40 mmHg SBP reduction: 1.92 mmHg, 95% CI: 2.40–1.43 mmHg DBP reduction: 1.27 mmHg, 95% CI, 2.34–0.20 mmHg
Bratton [51]	2015	51	4888	CPAP or MADs	DBP reduction: 2.5 mmHg, 95% CI: 1.5–3.5 mmHg DBP reduction: 2.0 mm Hg, 95% CI: 1.3–2.7 mmHg
Iftikhar [52]	2013	7	399	MADs	MBP reduction: 2.4 mmHg, 95% CI: 0.8–4.0 mmHg SBP reduction: 2.7 mmHg, 95% CI: 0.8–4.6 mmHg DBP reduction: 2.7 mmHg, 95% CI: 0.9–4.6 mmHg
Schein [53]	2014	16	1166	CPAP	Office SBP reduction: 3.20 mmHg, 95% CI: 1.72–4.67 mmHg Office DBP reduction: 2.87 mmHg, 95% CI: 0.55–5.18 mmHg Night-time SBP reduction: 4.92 mmHg, 95% CI: 1.14–8.70 Mean 24 h BP reduction: 3.56 mmHg, 95% CI: 0.33–6.79 mmHg Mean night-time BP reduction: 2.56 mmHg 95% CI: 0.68–4.43 mmHg
Fava [54]	2014	29	1820	CPAP	24- h SBP reduction: 2.6 ± 0.6 mm Hg 24 h DBP reduction: 2.0 ± 0.4 mm Hg
Labarca [55]	2021	10	606	CPAP	24 h SBP reduction: 5.06 mmHg, 95% CI: 2.13–7.98 mmHg 24 h DBP reduction: 4.21 mmHg, 95% CI: 1.93–6.50 mmHg Daytime SBP reduction: 2.34 mmHg, 95% CI: 2.27–6.94 mmHg Daytime DBP reduction: 2.14 mmHg, 95% CI: 0.67–4.96 mmHg Night-time SBP reduction: 4.15 mmHg, 95% CI: 1.29–7.01 mmHg Nighttime DBP reduction: 1.95 mmHg, 95% CI: 0.57–3.32 mmHg
Shang [56]	2022	19	1904	CPAP	SBP reduction: 5.01 mmHg 95% CI: 3.08–6.94 mmHg

Abbreviations: CI, confidence interval; CPAP, continuous positive airway pressure; DBP, diastolic blood pressure; HT, hypertension; MAD, mandibular advancement device; MBP, mean blood pressure; OR, odds ratio; OSA, obstructive sleep apnea; SBP, systolic blood pressure.

## Data Availability

Not applicable.
